# Left behind no more: ensuring equitable vaccine access to curb mpox in Africa

**DOI:** 10.1093/inthealth/ihaf018

**Published:** 2025-03-22

**Authors:** Obasanjo Bolarinwa, Aliu Mohammed, Yusuff Adebayo Adebisi, Oluwaseun Oyewo

**Affiliations:** Department of Public Health, York St John University, London, UK; Demography and Population Studies Programme, Schools of Public Health and Social Sciences, University of the Witwatersrand, Johannesburg, South Africa; Department of Health, Physical Education and Recreation, University of Cape Coast, Cape Coast, Ghana; College of Social Sciences, University of Glasgow, Glasgow, UK; Department of Medicine, Ladoke Akintola University of Technology, Ogbomoso, Nigeria

**Keywords:** Africa, equitable distribution, global health, health equity, mpox, vaccine rollout

Mpox, a zoonotic viral disease caused by the mpox virus (MPXV), has been endemic in Central and West Africa for decades, with transmission historically linked to animal-to-human contact and limited human-to-human spread.^1^ However, the 2022–2023 outbreak of clade IIb marked a turning point, with rapid global transmission predominantly via sexual contact, particularly among men who have sex with men (MSM) in non-endemic regions.^2^ Unlike past outbreaks, which were largely ignored by the global health community due to their localized nature, this outbreak spread across Europe, North America and South America, prompting immediate public health interventions, including targeted vaccination campaigns using smallpox-derived vaccines.^3^

Despite Africa's long-standing mpox burden, the response to this global outbreak once again exposed glaring inequalities in vaccine access. Countries in Europe and North America swiftly secured vaccines, leveraging their purchasing power and existing healthcare infrastructure, while African nations where mpox has been historically endemic were left behind.^4^ As of August 2024, >106 310 mpox cases have been recorded across 123 World Health Organization (WHO) member states, including 234 deaths.^5^ In Africa, 20 countries have reported cases, with the highest caseloads recorded in the Democratic Republic of the Congo (DRC), Burundi and Nigeria, yet vaccine access in these regions remains severely limited.^5^ The persistent disparities in vaccine distribution reflect the deep-rooted inequities in global health governance, where resource allocation continues to favour wealthier nations over regions with the greatest disease burden. The continuous spread of the virus led the WHO to declare it a public health emergency of international concern (PHEIC) on 14 August 2024.^6^ This declaration reinforces the urgent need for enhanced public health measures, particularly ensuring mpox vaccine equity, to curtail the increasing spread of the viral disease and minimize its burden globally.

Vaccination remains one of the most essential public health strategies for controlling infectious diseases.^7^ While the planned deployment of the mpox vaccine to affected African countries could potentially curtail the current spread of the disease, the success of the timely mpox vaccine rollout in Africa remains unpredictable. Multiple factors contribute to this uncertainty. First, vaccine supply remains limited, as production is primarily concentrated in high-income countries, with minimal commitments to ensuring equitable distribution. Second, healthcare infrastructure limitations, including inadequate cold chain storage, trained healthcare workers and last-mile distribution networks, pose significant barriers to widespread vaccine accessibility, particularly in rural and remote regions. Third, funding constraints further complicate vaccine acquisition, as many African nations rely on external funding sources rather than having independent procurement mechanisms. Finally, a lack of global coordination in mpox vaccine procurement mirrors the failures seen with the coronavirus disease 2019 (COVID-19), where vaccine nationalism left low- and middle-income countries at a severe disadvantage. Without urgent intervention, these barriers will continue to hinder Africa's ability to respond effectively to the mpox outbreak.

The COVID-19 pandemic exposed critical weaknesses in global health equity, many of which remain highly relevant to the mpox response. Both mpox and COVID-19 spread internationally, highlighting disparities in healthcare access and vaccine distribution. However, while COVID-19 is primarily airborne, mpox is transmitted mainly through close physical contact, with pre-existing smallpox-derived vaccines available but largely inaccessible in endemic African regions.^3^ The inequities observed during the COVID-19 vaccine rollout must serve as a lesson for improving the mpox response. COVID-19 saw wealthier nations monopolizing vaccine procurement, causing severe delays in access for African countries, a scenario that is now repeating with mpox. Additionally, the challenges of cold chain storage, healthcare worker shortages and last-mile vaccine delivery during the COVID-19 pandemic highlighted the need for innovative solutions, such as drone technology and mobile vaccination units, to improve accessibility, particularly in hard-to-reach communities.^8^

In the short term, measures for responding to the mpox outbreak must prioritize equitable vaccine access, efficient distribution logistics and community engagement. During the COVID-19 pandemic, wealthier nations monopolized vaccine procurement, leading to significant delays in access for African countries.^7^ To prevent a similar disparity, a globally coordinated framework should be implemented to ensure that mpox vaccines are distributed based on epidemiological need rather than financial capacity, with particular attention to ensuring that marginalized and high-risk populations are not excluded.^9^ Additionally, logistical barriers such as cold chain limitations and healthcare worker shortages, which hampered COVID-19 vaccine delivery in remote African regions, must be addressed.^7^ Innovative solutions, including drone technology and mobile vaccination units, could enhance accessibility to underserved populations. Equally crucial is community engagement, as vaccine hesitancy—exacerbated by misinformation and distrust of health authorities—was a major challenge during COVID-19 immunization efforts.^10^ Proactive public outreach and culturally tailored communication strategies are essential to fostering trust and ensuring the success of mpox vaccination campaigns.

While short-term measures focus on an immediate response, medium-term strategies must strengthen system resilience to ensure a more equitable and effective mpox response. One key priority is inclusive research and development, as seen during COVID-19, where vaccine trials were largely conducted in high-income countries, resulting in limited region-specific data for Africa.^8^ To avoid this gap, mpox vaccine research must actively involve African scientists and institutions, ensuring that clinical trials account for local epidemiological and genetic factors. Additionally, fairer global procurement mechanisms are essential to prevent the supply chain challenges that hindered initiatives like COVID-19 Vaccines Global Access, where wealthier nations prioritized their own populations, leaving African countries with insufficient access.^7^ Future mpox frameworks should establish guaranteed vaccine allocations for Africa, backed by stronger commitments from international donors to ensure timely and equitable distribution.

Long-term measures must focus on sustainable solutions that enhance Africa's ability to respond to future public health crises. One critical priority is building Africa's vaccine production capacity, as the continent's reliance on external suppliers significantly delayed COVID-19 immunization efforts.^8^ Without local manufacturing, African nations remain vulnerable to supply chain disruptions and global market competition. Investing in regional vaccine production facilities will not only ensure a more stable supply of mpox and other essential vaccines, but will also strengthen the continent's self-sufficiency in public health preparedness. Equally important is strengthening public health infrastructure, as weak healthcare systems and logistical barriers hindered the efficient rollout of COVID-19 vaccines in many African countries.^8^ To improve outbreak preparedness, sustained investments in healthcare infrastructure, supply chains and workforce development are necessary.

Despite repeated calls for global health equity, Africa continues to lack decision-making power in vaccine distribution, with high-income countries controlling both production and procurement. The mpox outbreak has once again exposed the structural inequalities in global health governance, where resource allocation favours the Global North, leaving endemic regions struggling for access. Unless long-term reforms are made to decentralize vaccine manufacturing and strengthen regional procurement mechanisms, Africa will continue to be sidelined in global health crises. A key solution to this power imbalance lies in shifting from donor-dependent vaccine supply chains to self-sufficient African-led manufacturing. The Partnership for African Vaccine Manufacturing, launched by the African Union, represents a step in the right direction, but stronger commitments from international donors and governments are necessary to accelerate progress. Without sustained investment in local capacity-building, vaccine nationalism will continue to dictate global health responses, leaving vulnerable populations behind.

The global response to mpox presents an opportunity to correct the mistakes made during the COVID-19 pandemic and build a more equitable system for vaccine distribution.

## Data Availability

All data are included in the article.
